# Crown rot in wheat: pathogen biology, host responses, and management strategies

**DOI:** 10.1007/s44154-025-00247-4

**Published:** 2025-08-25

**Authors:** Lefan Pu, Qiaojun Jin, Xuewei Cai, Chenfei Qu, Jiayi Zhang, Xingxuan Bai, Jia Guo, Zhensheng Kang, Jun Guo

**Affiliations:** State Key Laboratory for Crop Stress Resistance and High-Efficiency Production, Key Laboratory of Plant Protection Resources and Pest Management of Ministry of Education, College of Plant Protection, Northwest A&F University, Yangling, Shaanxi 712100 P. R. China

**Keywords:** Crown rot, *Fusarium*, Wheat, Pathogenic mechanism, Host responses, Integrated management

## Abstract

Crown rot (CR), caused by *Fusarium pseudograminearum* and related species, is a soil-borne disease threatening global wheat (*Triticum aestivum*) production, with yield losses exceeding 50% under severe infections. The rapid spread of CR in China, driven by straw retention policies and warming climates, highlights the need for interdisciplinary solutions. This review systematically integrates advances in CR research and addresses pathogen biology, host resistance, and sustainable management. Research on pathogen biology has clarified the distribution of major Fusarium species, the infection process, toxin profiles, mating types, and virulence factors. Host resistance to CR is quantitatively controlled, and through quantitative trait locus (QTL) mapping and omics-based approaches, several genes encoding transcription factors, receptor-like kinases and enzymes, signaling pathways and secondary metabolites involved in resistance have been identified. Advances in control strategies, including chemical and biological methods, as well as the application of nanotechnology, have shown promising results. The review also highlights future research directions, such as investigating the molecular mechanisms of pathogen-host interactions, identifying effectors and susceptibility genes for CR in wheat, and integrating multi-omics studies with high-resolution genetic maps to pinpoint CR resistance genes. These efforts are crucial for improving our understanding of the disease and developing effective management strategies.

## Introduction

Wheat is one of the most important crops globally, serving as a primary source of carbohydrates, proteins, and essential nutrients for human consumption. As the global population continues to grow, the demand for wheat production rises in parallel. However, wheat cultivation faces persistent threats from diseases and pests, with pathogenic fungal diseases being one of the most significant problems (Figueroa et al. 2018). Among the fungal diseases, crown rot (CR), caused by various Fusarium species, is a major detriment that impacts wheat and other cereal crops worldwide, particularly in arid and semi-arid regions (Akinsanmi et al. 2004). The disease targets the crown and root tissues of wheat, leading to browning, decay, and a reduction in the plant's ability to absorb water and nutrients, ultimately causing the distinctive"whitehead"appearance in severely infected plants (Kazan and Gardiner 2018).

CR can cause considerable yield losses in wheat, especially under drought conditions, where stressed plants become more vulnerable to the pathogen (Scherm et al. 2013). In Australia, CR is a significant concern, particularly in the northern wheat-growing regions. Yield losses in Australia due to CR range from 10 to 35% in moderately infected areas. In severe cases, losses can exceed 50% (Murray and Brennan 2009). On average, CR is estimated to cause approximately $80 million in annual losses to the Australian wheat industry (Murray and Brennan 2009). Similarly, in the Pacific Northwest region of the United States, CR has been reported to cause up to 35% yield loss in winter wheat (Smiley et al. 2005).

In China, CR has been reported in six provinces, including Henan, Jiangsu, Hebei, Anhui, Shandong, and Xinjiang (Zhang et al. 2015; Gao et al. 2024). In 2011, *Fusarium pseudograminearum* was identified as the emerging predominant pathogen causing CR in Henan province (Li et al. 2012). The disease has become an increasing threat to the Huang-Huai wheat-growing region (HHWGR), the main winter wheat cultivation area in China (Zhou et al. 2019). The rapid spread and development of CR in these regions have been attributed to farming practices like straw retention in the soil and the effects of global warming (Chakraborty et al. 2006). Yield losses in China due to CR are estimated to range from 10 to 30% annually (Zhang et al. 2020).

Beyond yield losses, CR also threatens food security due to the contamination of wheat stubble and grains with mycotoxins such as deoxynivalenol (DON) (Obanor and Chakraborty 2014) and nivalenol (NIV) (Monds et al. 2005). These toxins have harmful effects on both human and animal health (Mudge et al. 2006), adding another layer of concern to the management of this disease. Furthermore, the pathogens causing CR are diverse and complex in their combinations, and their distribution is influenced by climate and agricultural practices, which undoubtedly increases the difficulty of disease control.

As a soil-borne disease, CR remains difficult to control, and no highly effective methods have yet been developed. Developing and cultivating resistant varieties is the most efficient and environmentally friendly method to control crop diseases. However, no wheat germplasm exhibiting high resistance or immunity to this disease has been identified. While agronomic practices such as deep plowing, rotation with non-host plants, and straw burning can reduce the disease to a certain extent, these methods are neither economical nor environmentally sustainable. To develop effective and sustainable control strategies for CR, a deep understanding of the pathogen biology, its virulence factors, pathogen-host interactions, and host resistance is essential.

Using reverse genetics, several genes involved in the pathogenicity of *F*. *pseudograminearum,* the primary pathogen of CR, have been functionally characterized (Bi et al. 2024; Chen et al. 2021; Gardiner et al. 2012; Jiang et al. 2024; Kettle et al. 2015, 2016; Khudhair et al. 2020; Wang et al. 2022a; Wollenberg et al. 2019; Wu et al. 2024; Xia et al. 2021; Yang et al. 2024a; Zhang et al. 2023b). For the host side, researches on CR resistance have progressed in distinct phases: an initial peak between 2001 and 2010, followed by a decline from 2011 to 2015, then a renewed surge of interest since 2016. This trend reflects the growing recognition of CR as a global concern and the increasing engagement of research institutions. Among these, Chinese institutions have made the most significant contributions, followed by those in Australia, Canada, and the United States.

Meanwhile, multiple QTL associated with partial CR resistance (Table 1) have been mapped with forward genetics. Furthermore, transcriptomics, proteomics, and metabolomics studies have provided clues for the regulatory mechanisms of resistance-related genes, proteins, and metabolites (Powell et al. 2016; Qiao et al. 2021; Xu et al. 2024a, 2024b). This review systematically summarizes the advances on CR research, covering the pathogen biology including pathogen species, infection process, and virulence factors. It also highlights the host resistance mechanisms, including QTL loci, key resistance genes, and regulatory networks revealed by omics studies. Additionally, control strategies and future research directions were also discussed.

**Table 1:** QTLs for crown rot resistance in wheat and barley: germplasm sources, chromosomal loci (CHR), and phenotypic variance explained (PVE)

Resistant germplasm	Sources	Population type	Marker	CHR	QTL/Gene ID	Regions	PVE	Reference
Kukri	Kukri/Janz	DH	AFLP	4B	*Rht1*	*Mse1* CAC(139) -*Xgwm251*	48%	Wallwork et al. 2004
2–49	2–49/Janz	DH	SSR	1AL	-	Xwmc120 - Xwmc312	9%	Collard et al. 2005
				1DL	-	Xcfd19 - Xwmc216	21%	
				2AS	-	Xgwm339 - Xgwm425	2%	
				2BS	-	Xgwm388 - Xbarc349.1	4%	
				4BL	-	Xgwm251 - Xwmc349	4%	
				7BS	-	Xgwm400 - Xwmc476	6%	
W21MMT70	W21MMT70/Mendos	DH	SSR; AFLP	2B	-	Xgdm086 - Xgwm630	13–19.9%	Bovill et al. 2006
				2D	-	Xwmc018 - Xwmc190	4.8–10.2%	
				5D	-	Xbarc143 - Xbarc205	4.8–28.1%	
2–49; Gluyas Early	Gluyas Early/Janz	DH	SSR	1DL	*QCr.usq-1D1*	Xgwm337 - Xcfd65	20–21%	Collard et al. 2006
				2BS	*QCr.usq-2B1*	-	4–6%	
TX9425 (barley)	TX9425(barley)/Franklin (barley)	DH	DArT; SSR; AFLP	3H	*Qcrs.cpi-3H*	59.7 - 71.8 cM	34.1–60.4%	Li et al. 2009
CSCR6	Lang/CSCR6; Aus13832/CSCR6; Janz/CSCR6; Janz*2/CSCR6; Drysdale//Janz/CSCR6	RIL	DArT; SSR	3B	*Qcrs.cpi-3B*	32.8 - 39.8 cM	48.80%	Ma et al. 2009
				4B	*Qcrs.cpi-4B*	0.0 - 16.3 cM	22.80%	
Ernie	Batavia/Ernie	DH	DArT; SSR	3B	*-*	136.4 - 141.3 cM	34.60%	Li et al. 2010
2–49	2–49/W21MMT70	DH	DArT; SSR	1D	*QCr.usq-1D.1*	16.5 - 71.4 cM	10.40%	Bovill et al. 2010
				3B	*QCr.usq-3B.1*	145 - 176.7 cM	40.50%	
				7A	*QCr.usq-7A*	1.8 - 12.7 cM	3.80%	
Sunco; 2–49	Sunco/2–49	DH	DArT; SSR	1D	*QCr.usq-1D.1*	41.6 - 64.2 cM	-	
				2B	*QCr.usq-2B.2*	6.8–21.0 cM	8.40%	
				4B	*QCr.usq-4B.1*	0–30.3 cM	19.10%	
CSCR6	Lang/CSCR6	RIL	SSR	3B	*-*	-	14.70%	Yang et al. 2010
				4B	*-*	-	18.90%	
TX9425	Franklin/TX9425	DH		3H	*-*	-	24.90%	
Ernie	Ernie/Batavia	DH	DArT	3BL	*IhPt10505*	XwPt10505 - XwPt2277	35–49%	Liu et al. 2011
CSCR6	CSCR6/RIL	RIL	DArT; SSR	3BL	*-*	-	35–49%	
TX9425	TX9425(barley)/Franklin (barley)	DH	DArT; SSR	3HL	*4har*	near the centromere	34–63%	
CSCR6	CSCR6/Bellaroi	BC	SSR	6B	*-*	-	-	Ma et al. 2012
				4B	*-*	-	-	
	Aus13832/CSCR6; Lang/CSCR6	NIL		3B	*Rilm0181*	Xgwm0181	-	
	Janz*2/CSCR6	BC		3B	*Rcm0181*	Xgwm0181	-	
Sunco	Sunco/Macon; Sunco/Otis	RIL	DArT; SSR	3BL	*Qcrs.wsu-3BL*	Xgwm299 - Xgwm247 (1.8 cM)	36%	Poole et al. 2012
AWCS079	Baudin/AWCS079; Gairdner/AWCS079 RILF7; Franklin/AWCS079	RIL	DArT; SSR	1H	*Qcrs.cpi-1H*	XbPb-6065 - XbPb-8619	21.4–33.4%	Chen et al. 2013
				3H	*Qcrs.cpi-3H*	XbPb-6347 - XbPb-1183	28–39%	
EGA Wylie	EGA Wylie/Sumai3	RIL	DArT; SSR	5DS	*Qcrs.cpi-5D*	Xcfd189 - Xbacr	27–31%	Zheng et al. 2014
				2D	*Qcrs.cpi-2D*	1131013F0 - 1167665F0	16.5–20.2%	
				4B	*Qcrs.cpi-4B.1*	1241297F0 - 2304170F0	13.9–16.1%	
				4B	*Qcrs.cpi-4B.2*	1093616F0 - 2276137F0	15.6–18.7%	
	EGA Wylie/Chile; EGA Wylie/NK	RIL	DArT; SSR	5D	*Qcrs.cpi-5D*	-	22.2–29.6%	
				2D	*Qcrs.cpi-2D*	-	13.1–16.2%	
CSCR6	Janz*2/CSCR6; Lang/CSCR6	NIL	DArT	3B	*Gibberellin 2-beta-dioxygenase 8-like*	XwPt7514	-	Ma et al. 2014
2–49	2–49/Janz	DH	PCR; DArT	1AS	-	Xbarc148 - Xgwm164	12.8–16.4%	Martin et al. 2015
				1BS	-	Xcfd65 - Xgwm11	5.2–12.6%	
				1DL	-	Xcfd19 - Xwmc216	13.6–17.4%	
				2BS	-	Xgdm86 - Xcfa2278	8.80%	
				4BS	-	Xwmc467- Xgwm165	11.0–18.3%	
Sunco; 2–49	Sunco/2–49	DH	PCR; DArT	1AS	-	Xbarc148 - Xgwm164	3.3–7.2%	
				1BS	-	Xgwm11 - Xcfd65	4.4–7.2%	
				1DL	-	Xwmc216 - Xbarc162	6.6–12.6%	
				2BS	-	Xgwm630 - Xcfa2278	6.1–12.2%	
				3BS	-	Xgwm131 - XwPt-9310PCR	6.1–11.1%	
				4BS	-	Xwpt-7569PCR - Xwmc467	4–20.4%	
IRN497	IRN497/Janz	DH	PCR; DArT	2AL	-	Xgwm95 - Xcfa2043	19.70%	
				3BL	-	Xwmc236 - XwPt-0365PCR	18.80%	
				4BS	-	Xwmc467 - Xgwm165	13.80%	
				6DL	-	Xcfd188 - Xcfd47	5.1–7.1%	
CPI133814	CPI133814/Janz	DH	PCR; DArT	2DS	-	Xgwm484 - Xgwm102	6–12.1%	
				3AL	-	Xcfa2134 - Xcfa2262	9.9–11.9%	
				3BL	-	XwPt-0021PCR - Xgwm299	6.9–12.1%	
				6DL	-	Xbarc196 - Xbarc273	18.60%	
CSCS6	Aus13832/CSCS6; Janz*2/CSCR6; Lang/CSCR6	NIL	SSR	3B	*NBS-LRR; RPP13-like; RGA2*	CS3BLCR-04 - Xcfb3517 (1.5Mb)	-	Zheng et al. 2015
AWCS276	Baudin/AWCS276; Lockyer//AWCS276/AWCS079; Commander//AWCS276/AWCS079	NIL	SSR	4HL	*SilS6*	WMS6 - HVM67	40%	Habib, et al., 2016
CSCR6; EGA Wylie	EGA Wylie//Lang/CSCR6; EGA Wylie/3/EGA Wylie//EAG Wylie/Sumai3/4/CSCR6	RIL	SSR	3B	*Qcrs.cpi-3B*	-	49%	Zheng et al. 2017
				5D	*Qcrs.cpi-5D*		31.10%	
				2D	*Qcrs.cpi-2D*		20.20%	
AWCS276	Lockyer//AWCS276/AWCS079 (baley)	NIL	SSR	4H	*HORVU4Hr1G083930; HORVU4Hr1G084740; HORVU4Hr1G085140; HORVU4Hr1G085750*	WMS6 - HVM67	-	Habib et al. 2018; Jiang et al. 2019
-	Spring bread wheat lines	-	DArT	3B	*-*	XwPt-2193 - XwPt-2766	11.40%	Erginbas-Orakci et al. 2018
				2D	*-*	XwPt-669517	11.60%	
UC1110	UC1110/PI610750	Natural population; RIL	SNP	6A	*QFCR.heau-6A*	490486046 - 497462135（7.0 Mb）	7.77 - 15.16%	Yang et al. 2019
				2D	*QFCR.heau-2D*		7.15 - 9.29%	
				2A	*QFCR.heau-2A*		5.24 - 6.92%	
AWCS276	Fleet/AWCS799; Franklin/AWCS799 (baley)	RIL	SSR	6H	*Qcrs.caf-6H*	6H_453483214 - 6H_481998837	28.30%	Gao et al. 2019
2–49; Sunco	AUS29529/2/2–49/Cunningham//Kennedy/3/Sunco; CSCR16/2/2–49/Cunningham//Kennedy/3/Sunco/2*Pastor	MARS	SNP	1 A, 1B, 1D, 2D, 3B, 4 A, 4B, 5B, 6B, 7 A, 7B	-	-	-	Rahman et al. 2020
-	-	Diversity panel	SNP	2AL, 3AS, 3BS, 3DL, 4BS, 5BS, 5DS, 5DL, 6BS, 6BL, 6DS	-	-	-	Pariyar et al. 2020
-	-	Nested Association Mapping (NAM) population	DArT	6B	-	100.50 cm - 101.26 cm	-	Alahmad et al. 2020
-	04 Zhong 36/Liangxing 518	Association panel; RIL	SNP	5DL	*TraesCS5D01G138700.1, TraesCS5D01G141600.1, TraesCS5D01G143300.1*	218636512 - 232419393 bp (13.78 Mb)	14.59%	Jin et al. 2020
-	-	Pre-breeding population	SNP	1B, 2 A, 2B, 2D, 3 A, 3B, 3D, 4B, 5 A, 6 A, 6B, 7 A, 7B, 7D	-	-	-	Malosetti et al. 2021
-	AUS29529/Sunco/Pastor//Syn110	RIL	SNP	1 A, 1D, 2 A, 2B, 2D, 3 A, 3B, 4 A, 4B, 4D, 5 A, 5B, 5D, 6 A, 6B, 7 A, 7B	-	-	3.19 - 9.42%	Rahman et al. 2021
-	Yanzhan1 introgression lines; Bainong64/Jingshuang16; mutant library of Aikang 58		660 K SNP; 55 K SNP; Dcaps	4B	*TraesCS4B02G385200，TraesCS4B02G385500 *(*TaDIR-B1*)	664152928 - 664679942 bp (527 kb)	16.40–19.97%	Yang et al. 2021
Shishoumai	Shishoumai/Sanyuehuang	RIL	SNP	1B	*Qfcr.sicau.1B-4 *(*TraesCS1B03G1106600; TraesCS1B03G1088300*)	641360000–645130000 bp	39.66%	Hou et al. 2023a
AWCS799	AWCS799/Fleet; AWCS799/Franklin	NIL	SSR	6H	*Qcrs.caf-6H *(*HORVU.MOREX.r2.6HG0503730; HORVU.MOREX.r2.6HG0503700; HORVU.MOREX.r2.6HG0503660; HORVU.MOREX.r2.6HG0503840*.)	472.58 - 473.13 Mb (547 kb)	-	Gao et al. 2023a
C549	C549/3642; C549/Chuannong 16	RIL	SNP; SSR	2A	*Qfcr.cau-2A*	636896810 - 659575006 bp	24.42%	Xu et al. 2024b

## Pathogen biology

### Pathogen species

The historical records of Fusarium species causing root rot and CR in cereals date back to the early 1900s. Initially, the disease was attributed to a group of Fusarium species without precise identification but subsequent research identified several Fusarium species, notably *Fusarium pseudograminearum*, *F. culmorum*, and *F. graminearum* (Kazan and Gardiner 2018; Backhouse et al. 2004; Akinsanmi et al. 2004; Burgess et al. 1975; Özer et al. 2023; Scherm et al. 2013; Shikur Gebremariam et al. 2018). These species were identified as the primary culprits, with their prevalence varying by geographical location, local climate, and agricultural practices (Xu et al. 2018; Sabburg et al. 2015; Poole et al. 2013).

In the Pacific Northwest of the United States, *F. pseudograminearum* dominates in drier, warmer zones, while *F. culmorum* is more common in regions with higher moisture and cooler temperatures (Poole et al. 2013). Similarly, in Australia's dry and warm wheat-growing regions, *F. pseudograminearum* is the dominant CR pathogen, and *F. culmorum* prevails in the Victorian high/medium-rainfall region (Backhouse et al. 2004). A survey from 2009 to 2013 identified *F. asiaticum* of the trichothecene 3-acetyldeoxynivalenol (3-ADON) chemotype as the chief agent of CR, followed by *F. graminearum,* in five provinces of China (Jiangsu, Anhui, Henan, Hebei, and Shandong) (Zhang et al. 2015). *F. pseudograminearum* first appeared in Henan province in 2011, becoming the predominant CR pathogen in HHWGR (Henan, Shandong, Jiangsu, and parts of Shaanxi and Shanxi) from 2013 to 2016 (Zhou et al. 2019). The variation in predominant CR pathogens across China reflects differences in climate and farming practices. The study identified *F. asiaticum* as the most common CR pathogen collected samples from wheat-rice rotation areas with frequent rainfalls during wheat anthesis (Zhang et al. 2015). In Anhui and Jiangsu, where *F. graminearum* was dominant, warm conditions and moderate to high summer rainfall were prevalent (Zhou et al. 2019). The presence of *F. culmorum* in cooler and wetter regions, such as Xingtai and Handan in Hebei province (Zhou et al. 2019), aligns with earlier findings in the United States (Poole et al. 2013) and Australia (Backhouse et al. 2004). Thus, Fusarium species exhibit distinct environmental preferences that shape their geographic distribution. *F. pseudograminearum* predominates in warm, arid regions, while *F. graminearum* thrives in warm, humid conditions. In contrast, *F. culmorum* is better adapted to cooler, wetter climates. Studies in the North China Plain have demonstrated a positive correlation between *F. asiaticum* prevalence and annual precipitation levels (Xu et al. 2018). Additional species, such as *F. nygamai*, *F. acuminatum*, *F. equiseti*, *F. oxysporum*, *F. crookwellense*, and *F. chlamydosporum*, have also been associated with CR, though their roles are generally secondary to the primary pathogens (Zhang et al. 2023a; Özer et al. 2023; Backhouse et al. 2004).

Among these, *F. pseudograminearum* has emerged as the most consequential CR pathogen due to its aggressiveness and broad host range (Knight and Sutherland 2016). Climate change is amplifying its dominance—rising global temperatures correlate with increasing *F. pseudograminearum* prevalence in China and worldwide, displacing other Fusarium species in warmer regions (Bentley et al. 2008; Deng et al. 2020; Zhou et al. 2019). This trend underscores the urgency of species-specific identification for developing targeted resistance breeding programs and adaptive disease management strategies.

### Infection process of* F. pseudograminearum*

*F. pseudograminearum* and related species persist in crop residues (stubble) and soil as mycelium or chlamydospores (Kazan and Gardiner 2018). These survival structures can germinate and/or grow to produce asexual conidia, which act as the primary inoculum for new infections in subsequent growing seasons (Kazan and Gardiner 2018). The development of CR symptoms occurs gradually, and typically becomes visible 4 to 8 weeks after infection (Stephens et al. 2008; Knight and Sutherland 2016). Initial infection by the pathogen occurs in regions lateral to the basal leaf sheaths near ground level in field growing plants. The pathogen penetrates the epidermis via appressoria-like swelling at hyphal tips, targeting the lower crown, secondary root trace, and the sub-crown internode, often enters through natural openings like stomata. Initial lesions typically develop at guard cells, where specialized hook-like hyphae and appressorium-like foot structures enable penetration of the cell walls (Knight and Sutherland 2013). Once inside planta, the pathogen spreads both intercellularly and intracellularly, colonizing cortical tissues and sometimes re-emerging through stomata to initiate secondary infections. Some cell types, such as silica cells, resist fungal colonization, suggesting tissue-specific defense mechanisms (Knight and Sutherland 2013). Colonization of the parenchymatous hypodermis leads to cell discoloration and distortion, coinciding with visible discoloration used for disease rating. Continued fungal growth results in extensive tissue necrosis, particularly in the crown and basal stem regions (Knight and Sutherland 2016).

As the infection progresses, fungal hyphae spread and invade the vascular system, likely beginning at the vascular bundles in the nodes. This invasion disrupts the normal flow of water and nutrients within the plant, leading to drought-like symptoms, such as wilting and, in severe cases, the formation of white heads (Knight and Sutherland 2016). Histopathological sections reveal that xylem vessels become blocked by fungal mycelium and the tyloses (plant defense structures that block the xylem), further exacerbating this disruption. The pathogen travels from the stem base to the heads through the thin-walled parenchyma of the hypoderm and pith (Mudge et al. 2006). The upward hyphal growth through the pith is interrupted by the pith plexus, a single layer of thick-walled cells situated below the nodal anastomosis of vascular tissues. The vertical growing hyphae bypass this barrier by lateral growth from the peripheral cortex. Histological analysis shows significant degradation of cortical and vascular tissues, with evident browning and rotting in the infected areas. At least three lower internodes of the wheat plant are colonized by the fungus (Knight and Sutherland 2016). In partial-resistant genotypes, the fungi follow a similar infection pathway as in susceptible plants, but with slower progression (Knight and Sutherland 2016; Percy et al. 2012).

### Sexual reproduction of *F. pseudograminearum*

*F. pseudograminearum* completes its sexual cycle on infected plant debris, producing perithecia that contain ascospores (Bentley et al. 2008). However, the significance of ascospores as an inoculum source is uncertain, as perithecia are rarely observed in the field (Summerell et al. 2001). Unlike *F. graminearum*, *F. pseudograminearum* is heterothallic, requiring two distinct individuals with different mating types (*mat1-1* and *mat1-2*) for sexual reproduction (Francis and Burgess 1977). Although the teleomorph *Gibberella coronicola* is seldom documented (Aoki and O’Donnell, 1999), the ratio of mating types (*mat1-1*:*mat1-2*) is relatively balanced in populations from northeastern and southern Australia (Bentley et al. 2008), Tammin in Western Australia (Khudhair et al. 2019), and Henan in China (Zhang et al. 2024), suggesting that sexual reproduction in these regions is not limited by uneven mating type ratios.

However, in surveys conducted from 2014 to 2016 in Jiangsu and Shandong provinces of China, the ratio deviated significantly from 1:1, especially in Jiangsu (Deng et al. 2020). Skewed mating type frequencies were also reported in Karlgarin (Western Australia; Khudhair et al. 2019), Walgett (northeastern Australia), and Kedding (southwestern Australia; Bentley et al. 2008). Interestingly, perithecia were observed in Walgett (Bentley et al. 2008), indicating that sexual reproduction can still occur despite a skewed mating type ratio. Further research is needed to determine the factors influencing sexual reproduction in *F. pseudograminearum*.

The mating type frequency and distribution of a pathogen play critical roles in disease epidemiology (McDonald and Linde 2002). Regular monitoring of mating type frequencies and distribution is essential for effective CR disease control.

### Mycotoxins

Fusarium species produce a range of secondary metabolites, notably mycotoxins, which play a crucial role in the development and severity of diseases like CR. Among these mycotoxins, trichothecenes are the most significant group associated with CR, including DON and NIV (Deng et al. 2020). Trichothecenes inhibit protein synthesis in plant cells (Pestka et al. 2004) and weaken plant defense mechanisms, thus facilitating pathogen colonization and causing necrosis, which manifests as browning of the stem base and crown, characteristic symptoms of CR (Desmond et al. 2008a). While DON is a critical virulence factor for Fusarium Head Blight (FHB) infections (Bai et al. 2002), its role in CR is specific to fungal progression through the plant stem, though it is not required for the initiation of infection (Mudge et al. 2006; Tunali et al. 2012). Once produced in the infected stem base, DON can be transported to other parts of the plant, potentially leading to its accumulation in grains (Xu et al. 2024a).

Fusarium species are further classified into chemotypes based on the specific trichothecenes they produce. For example, DON chemotypes produce either 3-ADON or 15-ADON, while NIV chemotypes produce nivalenol (Ward et al. 2002). The geographical distribution of these chemotypes can impact disease management strategies. In Australia, 3-ADON is the predominant chemotype in *F. pseudograminearum* (Chakraborty et al. 2006; Khudhair et al. 2019), with 15-ADON first detected at low frequencies (~ 2.3% of isolates) in 2008 (Khudhair et al. 2019). In New Zealand, both 3-ADON and NIV chemotypes are present (Monds et al. 2005). In Sardinia, approximately 88% of *F. culmorum* isolates (the primary CR pathogen in the region) belong to the 3-ADON chemotype, with the remaining 12% being NIV (Balmas et al. 2015). Similarly, in Algeria, *F. culmorum* with the 3-ADON chemotype is the primary cause of CR (Laraba et al. 2017).

Geographic variation in chemotypes extends to China and Canada. In western Canada, three chemotypes of *F. pseudograminearum* have been identified (Clear et al. 2006). Similarly, in China, three chemotypes coexist (Deng et al. 2020; Zhang et al. 2024). However, unlike Australia, where 3-ADON predominates, 15-ADON is the main chemotype of *F. pseudograminearum* in Henan, China, which has both the largest wheat-growing area and the most severe CR outbreaks (Zhang et al. 2024). An earlier study found that 3-ADON was predominant in Jiangsu, whereas 15-ADON was more common in Shandong (Deng et al. 2020). A broader survey of Fusarium species causing CR in the main winter wheat regions (Jiangsu, Anhui, Henan, Hebei, and Shandong) revealed that *F. asiaticum* has two chemotypes (3-ADON and NIV), while *F. graminearum* only carries 15-ADON (Zhang et al. 2015). Overall, the distribution of Fusarium chemotypes in China follows a general pattern: 3-ADON isolates tend to dominate in warmer southern regions like Jiangsu, while 15-ADON isolates are more prevalent in cooler northern regions such as Shandong and Henan (Zhang et al. 2007; Shen et al. 2012).

The chemotype polymorphism in Fusarium species is maintained by balancing selection, which may impact pathogen fitness. However, in *F. graminearum*, 3-ADON and 15-ADON isolates exhibit similar virulence for FHB (Von Der Ohe et al. 2010; Shen et al. 2012), whereas DON chemotypes are more aggressive than NIV isolates (Shen et al. 2012). Whether different chemotypes vary in aggressiveness for CR remains unclear. Further research is needed to investigate the relationship between chemotype variations and pathogen fitness, as well as the factors driving the distinct regional distributions of 3-ADON and 15-ADON.

### Environmental factors

CR is significantly influenced by environmental conditions, including temperature, moisture, agronomic practices, and nutrient imbalances (Smiley et al. 2005; Smiley 2009). CR severity is particularly affected by precipitation levels and drought stress at the end of the wheat growing season (Smiley 2009). Wet conditions during the seedling stages, followed by water deficit toward the end of plant growth, favor CR development (Paulitz et al. 2002). Although drought stress may delay the initiation of infection, it ultimately accelerates pathogen proliferation and spread within the plant (Liu and Liu 2016). One possible reason is that drought stress promotes more frequent re-emergence of fungal hyphae from stomata, leading to the infection of neighboring cells. Additionally, drought stress increases the density and length of fungal aerial hyphae, which correlates positively with fungal biomass (Liu and Liu 2016). These findings highlight the complex interplay between environmental conditions and pathogen activity, underscoring the critical role of abiotic stress in exacerbating CR progression (Liu and Liu 2016).

Local climate conditions, such as precipitation and temperature, also affect the dominant pathogen species responsible for CR (Backhouse and Burgess, 2002). As previously mentioned, *F. culmorum* is more prevalent in cooler and wetter regions, while *F. pseudograminearum* dominates in drier and warmer zones (Poole et al. 2013; Backhouse, and Burgess, 2002). However, the presence of *F. pseudograminearum* in Australia (Backhouse, and Burgess, 2002) and the HHWGR of China (Zhou et al. 2019), suggests that its distribution is not strictly governed by climatic conditions. *F. graminearum*, meanwhile, is primarily found in warmer, wetter areas (Backhouse, and Burgess, 2002; Zhang et al. 2015; Zhou et al. 2019).

Interestingly, *F. pseudograminearum* has rapidly become the primary CR pathogen in China (Zhou et al. 2019). From 2009 to 2013, the predominant CR pathogens in China shifted from *F. asiaticum* and *F. graminearum* to *F. pseudograminearum*. One possible reason for this shift is a change in agronomic practices. In early 2010, the Chinese government banned straw burning after harvest for environmental protection. Consequently, stubble retention replaced straw burning, significantly increasing the inoculum load for CR. *F. pseudograminearum* has a high saprophytic fitness on wheat straw (Tunali et al. 2012), which likely contributed to its increased prevalence in China. Additionally, straw retention altered the soil microenvironment in ways that favored the survival of *F. pseudograminearum* (Deng et al. 2020). Crop rotation patterns further influenced the dominant pathogen species. In wheat-rice rotation areas, *F. asiaticum* was the dominant CR pathogen, whereas *F. pseudograminearum* was more prevalent in wheat–maize rotation regions in China (Zhang et al. 2015; Zhou et al. 2019).

### Virulence factors

Wheat produces the phytoalexin Benzoxazolinones (BOA) upon pathogen attack to inhibit the growth of pathogens. The genome of *F. pseudograminearum* contains a Fusarium detoxification of benzoxazolinone (FDB) gene cluster that is crucial for BOA detoxification. Within this cluster, *FDB2* (Kettle et al. 2015), encoding the *N*-malonyltransferase, and the zinc finger transcription factor gene *FDB3* are essential for BOA degradation and contribute to the virulence of *F. pseudograminearum* (Kettle et al. 2016).

*F. pseudograminearum* also produces Fusarium cytokinins in hyphae that are closely associated with living plant tissue during wheat infection (Sørensen et al. 2018; Blum, et al., 2019). These cytokinins likely plays a role in transitioning from the biotrophic to necrotrophic phase of the fungus, manipulating the infected plant tissue to act as a nutrient sink for the fungus (Sørensen et al. 2018; Blum et al. 2019). Interestingly, a subset of *F. pseudograminearum* isolates found exclusively in Western Australia carries an intact gene cluster responsible for the biosynthesis of fusaristatin A, a cyclic lipopeptide that unexpectedly reduces their aggressiveness (Wollenberg et al. 2019; Khudhair et al. 2020). The reason as to why these Western Australian isolates retain the fusaristatin A gene cluster remains unclear.

The genome of *F. pseudograminearum* has been well-characterized (Gardiner et al. 2012, 2018; Tai et al. 2023) with a detailed comparison to *F. graminearum* provided by Kazan et al. (2018). Their review highlights key similarities and differences in genome structure. With the availability of genome sequences, subsequent research has delved into the functional roles of specific genes. For example, comparative genomic analysis identified horizontally acquired genes *FpAH1* and *FpDLH1*, encoding an amidohydrolase and a dienelactone hydrolase, respectively, which are required for full virulence towards wheat and barley seedlings (Gardiner et al. 2012).

Reverse genetics approaches have characterized several transcription factors. The Zn2Cys6 transcription factors Fp487 and FpUME18 are required for vegetative growth, DON production, stress response, and virulence in *F. pseudograminearum*. Notably, Fp487 is a potential target for RNAi-based drugs (Yang et al. 2024a; Zhang et al. 2023b). Other transcription regulators like FpADA1 and FpLaeB play critical roles in asexual growth and virulence on wheat seedlings (Chen et al. 2020; Wu et al. 2023). However, individual deletion of these four genes also impacted growth and/or DON production, suggesting that their effect on virulence may be indirect, stemming from reduced growth and toxin production rather than directly affecting pathogenicity. For instance, deletion of the velvet protein gene *FpVelB* impaired virulence by controlling the expression of genes involved in secondary metabolism (Wu et al.  2024).

Peroxisome proliferator genes *FpPEX11a* and *FpPEX11b* (Wang et al. 2022a), as well as peroxisome biosynthetic receptor genes *FpPEX5* and *FpPEX7* (Bi et al. 2024), play pivotal roles in growth, conidiation and virulence. The endoplasmic reticulum luminal Hsp70 protein FpLhs1, involved in protein secretion, and the heat tolerance protein FpHsp104 are also critical for conidiation and pathogenicity (Chen et al, 2019; Xia et al. 2021).

Additional genes such as *FpZRA1* (involved in apoptosis), *FpBIR1* (an apoptosis-related gene), *FpHTF3* (a homeobox gene), *FpNPS9* (a non-ribosomal peptide gene), and *FpDEP1* (a component of the *Rpd3L* histone deacetylase complex) have been characterized through reverse genetics. Mutants lacking these genes exhibit reduced growth, decreased DON production, and significantly diminished virulence, underscoring their importance in the disease process (Jiang et al. 2024; Chen et al.  2021; Ma et al. 2022; Kang et al. 2020; Zhang et al. 2020).

Recent studies identified two effector proteins*,* Fp00392 (Yang et al. 2025a) *and* FpCDP1 (Liu et al. 2025), as conserved PAMPs (Pathogen-associated molecular patterns) in *F. pseudograminearum.* These proteins not only trigger immune responses in *Nicotiana benthamiana,* such as cell death, Reactive oxygen species (ROS) burst, and upregulation of defense-related genes, but are also critical for the pathogen’s full virulence. Gene knockout mutants (*ΔFp00392* and *ΔFpcdp1*) display significantly reduced pathogenicity on wheat (Yang et al. 2025a; Liu et al. 2025). This dual functionality of effectors, acting both as PAMPs and virulence factors, offers a novel perspective for studying *F. pseudograminearum*-wheat interactions.

**Fig. 1:**
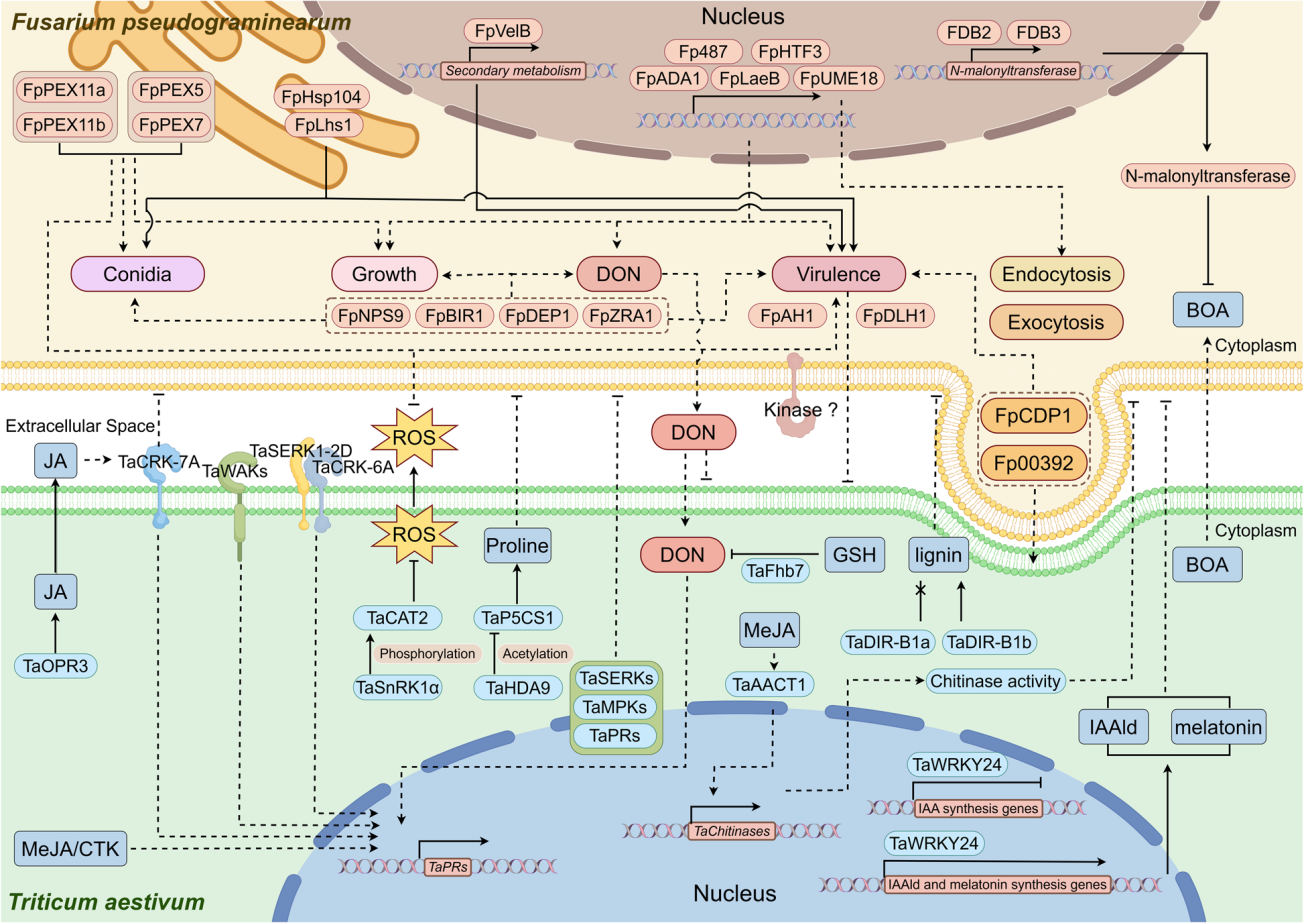
Schematic representation of the interaction between wheat (*Triticum aestivum*) and *F. pseudograminearum.* Solid lines represent well-characterized pathways; dashed lines indicate pathways requiring further investigation

These findings demonstrate the value of integrating genomic data with functional studies to identify and validate key pathogenicity determinants. However, many of the functionally characterized genes in *F. pseudograminearum* exhibit pleiotropic effects, making it difficult to discern whether their role in pathogenicity is direct or indirect (Fig. 1). Future research focusing on disentangling these relationships will be critical in advancing our understanding of this pathogen and in developing targeted strategies to manage CR in wheat.

## Host immunity

### CR resistance QTLs in wheat and barley and key germplasm resources

Partial CR resistance identified in wheat and barley is conferred by QTLs (Table 1). To date, 128 QTLs associated with CR resistance have been identified, distributed across 19 wheat and 4 barley chromosomes. Of these, 120 are located on wheat chromosomes (Table 1), and 8 on barley chromosomes. In wheat, the B genome contains the highest number of QTLs, with chromosome 3B harboring 20 QTLs (16.67% of the total). The QTLs on 3B have substantial effects, explaining up to 49% of the observed resistance phenotype (Ma et al. 2010, 2014; Li et al. 2010; Yang et al. 2010; Liu et al. 2011; Poole et al. 2012; Zheng et al. 2015, 2017; Erginbas-Orakci et al. 2018). Candidate resistance-related genes on 3B include *Nucleotide-binding site-leucine-rich repeat* (*NBS-LRR*), *Recognition of Peronospora Parasitica 13-lik*e (*RPP13-like*), *Rho GTPases activating protein 2* (*RGA2*), and the gibberellin metabolism-related gene *GA2oxs8-like*. Chromosome 4B also plays a significant role in CR resistance, harboring 16 QTLs that explain up to 35% of phenotypic variation (Chalmers et al. 2001; Wallwork et al. 2004; Pariyar et al. 2020; Rahman et al. 2020, 2021; Malosetti et al. 2021; Yang et al. 2021). Key candidate genes on 4B include the dwarfing gene *Rht-1* and the lignin biosynthesis gene *TaDIR-B1*. In barley, 8 QTLs have been identified across 4 chromosomes. Chromosome 3H harbors 4 QTLs, explaining up to 28% of the phenotypic variation (Li et al. 2009; Yang et al. 2010; Liu et al. 2011; Chen et al., 2013). 2 QTLs have been mapped to chromosome 4H (Habib et al. 2016, 2018; Jiang et al. 2019). A single QTL each was identified on chromosomes 1H and 6H, each explaining over 20% of the phenotypic variation (Chen et al. 2013; Gao et al. 2019, 2023a). Additionally, research by Wang et al. (2020) published in Science revealed that the *Fhb7* gene, derived from the 7E chromosome of *Thinopyrum ponticum* and encoding a glutathione S-transferase (GST), not only confers resistance to FHB, but also provides effective resistance to CR. Wheat-*Thinopyrum ponticum* translocation lines carrying *Fhb7* have shown high levels of CR resistance in field trials. This discovery underscores the importance of utilizing genetic resources from heterologous species.

Frequently used wheat germplasms for CR resistance include: CSCR6 (Ma et al. 2010, 2012, 2014; Yang et al. 2010; Liu et al. 2011; Zheng et al. 2015), 2–49 (Collard, et al., 2005, 2006; Bovill et al. 2010; Martin, et al., 2015; Rahman et al. 2020), Sunco (Bovill et al. 2006; Poole et al. 2012; Martin et al. 2015; Rahman et al. 2020), and Ernie (Li et al. 2010; Liu et al. 2011). Among these, CSCR6 is strongly linked to QTLs on chromosome 3B, while populations derived from 2–49 consistently map QTLs to chromosome 1D. In barley, the primary source of CR resistance is AWCS276 (Habib et al. 2016, 2018; Jiang et al. 2019; Gao et al. 2019) and TX9425 (Li et al. 2009; Yang et al. 2010). AWCS276 is strongly linked to QTLs on chromosome 6H (*Qcrs.caf-6H*) (Gao et al. 2019), while populations derived from TX9425 consistently map QTLs to chromosome 3H (*Qcrs.cpi-3H*) (Li et al. 2009).

### Application of omics in the study of CR resistance

Omics studies provide a comprehensive molecular basis for understanding CR resistance mechanisms in wheat and barley. Early microarray studies showed that *F. pseudograminearum* infection induced the expression of 1,248 wheat genes while repressing 1,497, with resistant wheat varieties exhibiting a faster and more robust transcriptional response compared to susceptible ones (Desmond et al. 2008b). RNA-seq analysis further identified over 2,700 differentially expressed genes (DEGs) in hexaploid wheat following *F. pseudograminearum* infection. Notably, homologous genes in the B and D genomes displayed particularly strong responses, highlighting their potential roles in resistance (Powell et al. 2017b). Using transcriptome analysis of near-isogenic lines, Su et al. (2024) found that while gene expression networks differ under drought stress at various time points, *F. pseudograminearum* infection and drought stress activate similar regulatory frameworks. These findings suggest the overlapping of signaling and metabolic pathways in response to biotic and abiotic stresses. The application of transcriptomics has also extended to model species. For example, 2,498 DEGs were identified in *Brachypodium distachyon* during *F. pseudograminearum* infection, showing significant similarity to those in wheat. However, differences in the production of specific defense metabolites between *B. distachyon* and wheat emphasize the importance of caution when extrapolating findings from model species to wheat-* F. pseudograminearum* interactions, as inaccurate conclusions may arise (Powell et al. 2017a).

Label-free quantitative proteomics have been employed to uncover changes in wheat upon *F. pseudograminearum* infection. For instance, Jin et al. (2021) identified 783 differentially expressed proteins (DEPs) following *F. pseudograminearum* infection, which were primarily associated with pathways related to metabolism, cellular processes, and defense responses. Similarly, quantitative proteomics analysis using tandem mass tags (TMT) identified 366 DEPs between resistant and susceptible wheat varieties (Qiao et al. 2021). These proteins are linked to critical processes such as plant hormone signaling, photosynthesis, and the synthesis of secondary metabolites. Additionally, a protein interaction network constructed in the same study highlighted the dual roles of sugar metabolism- and photosynthesis-related proteins. These proteins not only supply energy for defense responses but also participate in signal transduction and regulate the activation of defense-related genes (Qiao et al. 2021).

Metabolomic analyses have demonstrated that CR significantly alters the production of defense-related metabolites in wheat, including the accumulation of key compounds such as tryptamine and serotonin, which play essential roles in defense responses (Powell et al. 2016). Xu et al. (2024a) reported significant differential enrichment of metabolites in pathways such as tryptophan metabolism, phenylpropanoid biosynthesis, and ascorbate and linoleic acid metabolism pathways, following *F. pseudograminearum* infection, through metabolomic profiling. Notably, two key metabolites in the tryptophan metabolism pathway, indole-3-acetaldehyde (IAAld) and melatonin, showed substantial accumulation during *F. pseudograminearum* infection (Xu et al. 2024c). The study further revealed that exogenous application of IAAld and melatonin significantly enhanced wheat resistance to *F. pseudograminearum* (Xu et al. 2024c). In addition, *F. pseudograminearum* infection significantly alters the levels of jasmonic acid (JA), salicylic acid (SA), cytokinins (CTKs), and auxins (Gao et al. 2023b). Interestingly, pre-sowing seed treatments with JA and SA solutions were shown to enhance CR resistance of wheat, likely by priming defense responses of the plants (Gao et al. 2023b). These omics studies offer valuable clues for understanding CR resistance in wheat. However, the integration of multi-omics data can contribute to the development of a comprehensive disease-resistance network, providing deeper insights into plant responses to pathogen attacks (Tang et al. 2021). For instance, recent multi-omics research integrating transcriptomic, proteomic, and metabolomic data in common wheat has systematically decoded the impact of transcriptional regulation and post-translational modifications on protein abundance, along with complex functional interactions between genes (Zhang et al. 2025). This investigation identified a critical protein module, TaHDA9-TaP5CS1, which influences wheat resistance to CR through acetylation modifications (Zhang et al. 2025). TaHDA9 (histone deacetylase) regulates the expression of TaP5CS1 (a key enzyme in proline synthesis), forming a precisely coordinated regulatory network (Zhang et al. 2025). Therefore, future research should prioritize multi-omics approaches to further explore the mechanisms underlying the *F. pseudograminearum*-wheat interaction.

### Resistance genes and their mechanisms

In recent years, receptor-like kinase (RLK) genes have been identified as key regulators of wheat resistance to CR. For instance, TaCRK-7A, a cysteine-rich receptor-like kinase, has been shown to inhibit pathogen growth while enhancing the expression of defense-related genes through the JA signaling pathway (Wu et al. 2021). Similarly, TaRLK-6A was recently identified as a key RLK that interacts with TaSERK1-2D to regulate the expression of defense-related genes, thereby enhancing overall disease resistance (Qi et al. 2024). Members of the wall-associated kinase (WAK) family, TaWAK-6D and TaWAK-5D600, also play significant roles in resistance to CR (Qi et al. 2021, 2023) by regulating resistance through the induction of critical defense-related genes, including *TaSERK1*, *TaMPK3*, and *TaPR1* (Qi et al. 2023).

Transcription factors play crucial roles in regulating disease resistance. One notable example is the transcription factor TaWRKY24, which enhances CR resistance by modulating tryptophan metabolism (Xu et al. 2024c). TaWRKY24 suppresses the synthesis of indoleacetic acid (IAA) while promoting the accumulation of IAAld and melatonin. This regulation links transcriptional control of IAA to the metabolic defense pathways (Xu et al. 2024a), providing a novel perspective on how transcription factors contribute to plant resistance.

Enzymes, along with receptor kinases and transcription factors, also play critical roles in defending *F. pseudograminearum* infection. *TaAACT1*, a cytosolic acetoacetyl-CoA thiolase gene in wheat, plays a crucial role in disease resistance. Silencing *TaAACT1* using virus-induced gene silencing (VIGS) significantly reduced wheat resistance to *F. pseudograminearum* and exacerbated disease symptoms (Xiong et al. 2023). In wheat plants with silenced *TaAACT1*, the transcript levels of defense-related genes, including chitinases (e.g., *TaChitinase 2*, *TaChitinase 3*, *TaChitinase 4*) and defensins (*TaDefensin*), were significantly reduced. Furthermore, the expression of *TaAACT1* was markedly induced by exogenous methyl jasmonate (MeJA) treatment (Xiong et al. 2023), suggesting the involvement of *TaAACT1* in JA-mediated CR resistance. ROS scavenging systems also play vital roles in wheat disease resistance. Recent studies have demonstrated that the wheat catalase gene *TaCAT2* enhances resistance to CR by eliminating ROS (Yang et al. 2025b). More significantly, the protein kinase TaSnRK1α phosphorylates TaCAT2, substantially increasing its stability and activity, thereby establishing a novel regulatory model that elucidates the crucial role of post-translational modifications in modulating wheat disease resistance (Yang et al. 2025b).

### Plant hormones and metabolite regulation network

Plant hormones play a central role in CR resistance. Gao et al. (2023b) reported that *F. pseudograminearum* infection induces significant changes in plant hormones, particularly JA, SA, CTKs, and IAA. Among these, JA exhibited the strongest role in promoting resistance, as soaking seeds in JA solution significantly enhanced wheat resistance to CR. Wu et al. (2021) further demonstrated that exogenous JA application upregulated the receptor-like kinase TaCRK-7A, which enhanced resistance by promoting the expression of defense-related genes, suggesting that JA signaling may be mediated through TaCRK-7A. Overexpression of the JA biosynthesis gene *TaOPR3* not only enhanced wheat resistance but also promoted root and stem growth (Gao et al. 2023b), demonstrating JA's dual potential in coordinating plant developmental processes and maintaining growth-defense equilibrium. In addition, the JA derivative MeJA can activate the expression of defense-related genes, further emphasizing the positive role of JA in CR resistance of wheat. Interestingly, benzothiadiazole and DON also activate defense gene expression (Desmond et al. 2008a, 2008b), suggesting their potential involvement in wheat CR resistance. In contrast, treatments with CTKs increased wheat susceptibility to CR (Gao et al. 2023b). Moreover, *F. pseudograminearum* infection was found to activate the tryptophan and tyrosine biosynthetic pathways, leading to the accumulation of tryptamine and serotonin (Powell et al. 2016), highlighting possible roles of these metabolites in *F. pseudograminearum*-wheat interaction.

Collectively, these findings provide valuable insights into the metabolite-mediated mechanisms underlying CR resistance in wheat and barley, offering potential targets for enhancing resistance. They also establish a theoretical foundation for developing novel resistance strategies (Fig. 1). Future research should aim to evaluate the interactions between distinct regulatory networks by combining metabolite profiling with gene regulatory studies, and translate these insights into practical applications for improving crop resilience.

### Prevention and control strategies for CR

As no wheat germplasms are currently available that are immune or highly resistant to CR, control of the disease mainly relies on cultivating moderately resistant varieties, agronomy approaches and chemical control. *Fusarium* spp., overwintering in soil and crop residues, are the primary inoculum, with disease severity directly linked to soil pathogen concentration. Straw retention in the field exacerbates CR by increasing the primary inoculum, while straw burning reduces CR but harms soil health by decreasing organic carbon, moisture content, and soil biological activity. Crop rotation with non-host plants such as annual alfalfa (*Medicago* spp.), canola (*Brassica napus*) and lupin (*Lupinus* spp.) effectively reduce CR by altering the soil microbial communities (Theron et al. 2023) and enhance residue decomposition. Soil conditions also influence CR severity. Deep plowing enhances soil structures, improves permeability, reduces pathogen load, supports root growth, and therefore reduces CR severity. Adequate zinc supplementation mitigates CR severity (Grewal et al. 1996), whereas excessive nitrogen application intensifies it, possibly by causing water stress (Davis et al. 2009).

In the absence of highly resistant or immune wheat varieties, chemical fungicides are vital for CR management. Pre-sowing seed treatments with azoxystrobin (Guo et al. 2022), pyraclostrobin (Hou et al. 2023b), cyclobutrifluram (Sun et al. 2024), phenamacril (Hou et al. 2023c), tebuconazole (Liu et al. 2024b), fludioxonil (Zhang et al. 2022b; Chen et al. 2024), difenoconazole (Zhang et al. 2022b) and fluazinam (Luo et al. 2024) have been shown to effectively inhibit CR in field trials. Additional fungicides such as metconazole (14α-demethylation inhibitor, DMI) (Liu et al. 2023) and hexaconazole-lentinan (LNT) (Yang et al. 2022) have shown potential in greenhouse trials. However, only limited formulations, such as fludioxonil × clothianidin and prothioconazole × tebuconazole are registered in China, underscoring the need to screen and register additional effective fungicides.

Nanotechnology represents an alternative to chemical fungicides. For example, nanochitin whisker suspension (NCs) enhances wheat growth and inhibits the growth of *F. pseudograminearum* and *F. graminearum* (Xue et al. 2018) through synergistic effects (Liang et al. 2018). Additionally, silver nanoparticles (Ag-NPs) and zinc nanoparticles (Zn-NPs), show promise in controlling CR and boost wheat yields (Masmoudi et al. 2023; Al-Quwaie et al. 2024). Combining nanotechnology with chemical fungicides offers a novel and sustainable approach to CR management.

Biological control, compared to chemical control, has a milder impact on the environment and significantly contributes to sustainable agriculture. Various biocontrol microorganisms with effective capabilities against CR have been identified (Table 2). Plant-growth-promoting rhizobacteria (PGPR) are rhizosphere bacteria that inhibit pathogens by competing for space and nutrients, producing antimicrobial compounds, and promoting growth and inducing systemic resistance in plants (Bhattacharyya et al. 2012). For example, *Pseudomonas* spp. proliferate rapidly to out-compete pathogens (Bonaterra et al. 2022). *P. cepacia* A3R can reduce CR caused by *F. graminearum* when applied via seed dressing or soil drenching (Huang and Wong 1998). Similarly, *P. mediterranea* HU-9 (Ullah et al. 2020) and *P. plecoglossicida* (Makhlouf et al. 2024) effectively suppress CR caused by *F. culmorum or F. graminearum* or *F. pseudograminearum.* Additionally, *Lysobacter* spp. produce antibiotics that suppress pathogens. The spore suspension of *L. antibioticus* HS124 efficiently inhibits CR and promotes wheat growth through seed soaking or seedling irrigation (Monkhung et al. 2016; Kim et al. 2019). Likewise, seed treatments with *B. amyloliquefaciens* YB-161 (Lin et al. 2020), *B. subtilis* YB-15 (Xu et al. 2022), *B. velezensis* YB-185 (Zhang et al. a), *B. amyloliquefaciens* ZK-9 (Yi et al. 2024) or *B. tequilensis* YB-1145 (Liu et al. 2024a) reduces CR while promoting plant growth. Seedling irrigation with *B. halotolerans* QTH8 or *B. siamensis* YB-1631 has similar effects (Dong et al. 2023). *Paenibacillus polymyx* SGK2 competes for ion via siderophores production and mitigates CR triggered by *F. culmorum* and *F. graminearum* (Lounaci et al. 2017). Furthermore, *Streptomyces* spp., the largest branch of Actinobacteria, are widely present in soil and the plant rhizosphere and can suppress soil-borne diseases through nutrient competition, systemic resistance induction, and antibiotic production (Winter et al. 2019). Soil treatment with *Streptomyces* S05/S06 (Winter et al. 2019) and seed coating with *Streptomyces* MH71/MH243 (O’Sullivan et al. 2021) significantly reduce CR.

**Table 2:** Recently reported biocontrol agents against *Fusarium* spp. and their action mechanisms

Typle	BCAs		Strains	Action mechanism	Reference
Bacteria	*Stenotrophomonas* spp.	*S. rhizophila*	SR80	Induced host resistance	Liu et al. 2021
	*Pseudomonas* spp.	*P. cepacia*	A3R	—	Huang and Wong 1998
		*P. mediterranea*	HU-9	Antagonism; Induced host resistance	Ullah et al. 2020
		*P. plecoglossicida*	NR_114226	Produced chitinase, protease, hydrocyanic acid, indole acetic acid and siderophore	Makhlouf et al. 2024
	*Lysobacter *spp.	*L. antibioticus*	HS124	Produced volatile organic compounds (VOCs)	Kim et al. 2019
	*Bacillus* spp.	*B. amyloliquefaciens*	YB-161	Inhibited mycelial growth	Lin et al. 2020
		*B. subtilis*	YB-15	Antagonism; Induced host resistance	Xu et al. 2022
		*B. velezensis*	YB-185	Inhibited mycelial growth and conidial germination	Zhang et al. 2022a
		*B. amyloliquefaciens*	ZK-9	Produced lipopeptides	Yi et al. 2024
		*B. tequilensis*	YB-1145	Inhibited mycelial growth	Liu et al. 2024a
		*B. siamensis*	YB-1631	Inhibited mycelial growth and conidial germination; Induced host resistance	Dong et al. 2023
	*Paenibacillus *spp.	*P. polymyx*	SGK2	Competed for iron	Lounaci et al. 2017
	*Streptomyces *spp.	—	S05/S06	Antagonism and effective competition	Winter et al. 2019
		—	MH71/MH243	Inhibited fungal growth	O’Sullivan et al. 2021
Fungi	*Trichoderma* spp.	*T. afroharzianum*	—	—	Stummer et al. 2020
		*T. atroviride*	HB20111	Changed the composition and structure of the fungal community	Sui et al. 2022
		*T. harzianum*	Tr906	Suppressed pathogen abundance in planta	Stummer et al. 2022
		*T. gamsii*	A5MH		Stummer et al. 2022
		*T. longibrachiatum*	TG1	Induced host resistance	Boamah et al. 2021
	*Chaetomium* spp.	*C. globosum*	12XP1-2–3	Competition for nutrients and ecological niches	Feng et al. 2023
	*Talaromyces* spp.	*T. muroii*	TM28	Antagonism	Yang et al. 2024b
	*Piriformospora* spp.	*P. indica*	——	Induced host resistance; Induced the phenylpropanoid pathway	Dehghanpour-Farashah et al. 2019 ; Li et al. 2023

Certain fungi also exhibit biocontrol functions. *Trichoderma* spp. suppress CR through hyperparasitism, systemic resistance induction, secondary metabolite production, and secretion of cell wall-degrading enzymes (Stummer et al. 2020). Seed treatments with *T. afroharzianum*, *T. atroviride*, *T. harzianum*, *T. gamsii*, and *T. longibrachiatum* effectively reduce CR severity (Stummer et al. 2020; Dendouga et al. 2016; Kthiri et al. 2020; Stummer et al. 2022; Boamash et al. 2021; Sui et al. 2022). *Chaetomium globosum* 12XP1-2–3 delays CR onset, decrease the white ear rate and disease index, and enhance yield through nutrition competition (Feng et al. 2023). *Talaromyces muroii* TM28 exhibits antagonism against *F. pseudograminearum* (Yang et al. 2024b). Additionally, *Piriformospora indica* induces systemic resistance via phenylpropanoid pathway, and enhances wheat resilience to *F. pseudograminearum* (Dehghanpour-Farashah et al. 2019; Li et al. 2023). However, despite promising results, biocontrol agents face challenges in field application, including variability in effectiveness due to environmental conditions and unsteady vitality. Consequently, future research should prioritize strategies to stabilize efficacy, such as enhancing microbial formulations and developing environmental adaptation techniques.

## Conclusions and future perspectives

Significant progress has been made in understanding CR in recent years, particularly in areas such as pathogen biology, host resistance, and control strategies. However, critical challenges remain in deciphering pathogen infection mechanisms, resistance resource development, pathogen-host interactions, and establishing sustainable control approaches.

### Pathogen infection mechanisms

Although multiple genes associated with *F. pseudograminearum* pathogenicity (e.g., *FDB2*, *FpVelB*, *FpZRA1*) have been identified, their roles often involve pleiotropic effects, and their precise molecular functions during infection remain poorly defined (Zhang et al. 2020; Wu et al. 2024). Notably, recent studies have characterized two CR-specific secreted proteins, Fp00392 and FpCDP1, which play dual roles as both major virulence factors and PAMPs (Yang et al. 2025a); Liu et al. 2025). However, these studies did not fully elucidate how these effectors balance their dual roles as virulence factors and PAMPs. Despite these advances, the broader landscape of CR-specific effectors remains largely unexplored. Further research is needed to determine whether other CR pathogens secrete lineage-specific effectors that can modulate wheat immune responses, which will require comprehensive approaches such as host target screening technologies (Kazan and Gardiner 2018).

Histological studies using microscopy have provided preliminary insights into the infection dynamics of *F. pseudograminearum* (Knight and Sutherland 2013, 2016; Percy et al. 2012). However, these studies are limited by non-consecutive observation timepoints and rely primarily on fluorescence microscopy. To gain a comprehensive view of infection, future research should integrate confocal microscopy, transmission electron microscopy, scanning electron microscopy, along with spatiotemporal resolved transcriptomics approaches such as single-cell sequencing (Tang et al. 2023).

### Pathogen-host interaction mechanisms

Early recognition signals are crucial for successful pathogen establishment. A study on *F. graminearum* has shown its ability to sense host roots through diffusible signals (Ding et al. 2020). In parallel, the"stealth"strategies by which pathogens dynamically regulate virulence factors (e.g., CTKs) to evade host immunity require further exploration (Sørensen et al. 2018). At the effector-host interaction level, mechanisms by which effectors target host proteins are emerging as a critical area of focus. For example, in wheat FHB, the effector FgEC1 promotes infection by suppressing host ROS burst (Shang et al. 2024), but whether similar effectors exist in CR pathogens remains to be explored. Simultaneously, how host resistance proteins (e.g., receptor kinase TaCRK-7A; Wu et al. 2021) recognize pathogen elicitors and activate immune-related signaling pathways also deserves in-depth investigation.

### Resistant resource development

Current CR resistance breeding primarily relies on partial resistance QTLs, as no wheat genotype with complete immunity has been discovered (Zheng et al. 2017; Yang et al. 2021). Expanding the search for resistant alleles to wild relatives and global wheat germplasm repositories is key to discovering new resistance sources. For example, emmer wheat (*Triticum dicoccoides*) may harbor unexplored resistance traits (Malosetti et al. 2021). Furthermore, the model species like *Brachypodium distachyon* inhibits pathogen entry at pre-contact stage by secreting defensive metabolites (Ding et al. 2021). Elucidating their biosynthetic pathways may provide valuable insights for developing novel strategies to control CR. Introducing non-host resistance genes into wheat, via resistance gene pyramiding and transgenic technology, holds promise for enhancing disease resistance (Powell et al. 2017a). CRISPR-Cas9 tools enable further targeted resistance improvement, exemplified by editing susceptibility genes like *TaDIR-B1* (Yang et al. 2021). Examples from other pathosystems, such as *TaPsIPK1* editing for stripe rust resistance (Wang et al. 2022b), highlight the potential for these technologies in CR management.

### Seedling vs. adult-plant resistance assessment: ongoing debate and integration

A key debate in CR research concerns whether to prioritize seedling-stage or adult-plant (field) resistance assessment. Seedling assays offer rapid, high throughput, cost-effective screening, making them valuable for large-scale genetic studies and early-stage resistance identification (Wildermuth and Mcnamara 1994; Li et al. 2009, 2022; Erginbas-Orakci et al. 2016). Although seedling-stage assessments effectively control both biotic and abiotic variables, the resistance traits identified under these conditions sometimes show discrepancies when tested in field settings with complex interacting factors. (Hogg et al. 2007; Zheng et al. 2014). In contrast, adult-plant evaluations better reflect yield impact and agronomic performance (Hogg et al. 2007; Liu and Ogbonnaya 2015; Ma et al. 2010). However, adult-plant evaluations are time-consuming and may miss potential resistance sources due to environmental masking of phenotypes.

Seedling- and adult-plant resistance are only moderately correlated (r = 0.43—0.63; Zheng et al. 2015), likely due to stage-specific gene/QTL expression and genotype–environment interactions (Bovill et al. 2006; Yang et al. 2010; Shi et al. 2020; Liu and Ogbonnaya 2015). Nonetheless some loci (e.g., the 3BL QTL) consistently confer resistance at both stages in certain cultivars (Collard et al. 2005; Martin et al. 2015; Rahman et al. 2020; Pariyar et al. 2020), highlighting the complementary of the two approaches.

Common seedling inoculation protocols—such as spore droplets application, seed soaking, or moist towel methods—are well suited for large-scale screening, but cannot mimic field conditions (Mitter et al. 2006; Li et al. 2008, 2022; Yang et al. 2010; Smiley 2019). In contrast, adult-plant methods—including colonized grain/substrate and infested-soil inoculation—offer greater ecological relevance, though they require careful control of inoculation uniformity and environmental factors (Hogg et al. 2007; Wildermuth and Mcnamara 1994; Poole et al. 2012; Malosetti et al. 2020).

In conclusion, researchers should select assessment stages according to their specific experimental objectives and resources. Alternatively, both the seedling assay and field (or adult-plant) evaluation can be strategically combined: seedling assays enable early elimination of susceptible genotypes, while field evaluations validate resistance under realistic conditions. This flexible, integrated approach has the potential to strengthen breeding programs and advance CR research in wheat.

### Sustainable control

Numerous biocontrol microorganisms including *Bacillus velezensis* YB-185 and *Trichoderma harzianum* have been shown to have efficacy against CR. However, their field performance is significantly influenced by environmental factors (Liu et al. 2024a; Stummer et al. 2020; Zhang et al. 2022a). Microbial application engineering provides new approaches that combine biocontrol microorganisms with nanomaterials (e.g., chitosan nanocrystals) can enhance strain colonization efficiency and stress tolerance (Liang et al. 2018). Future research should prioritize enhancing microbial environmental adaptability through gene editing, such as introducing drought-resistant and salt-tolerant genes (Al-Quwaie et al. 2024). Moreover, the integration of metabolomic profiling of antimicrobial metabolites, coupled with AI-driven analysis and molecular docking offers promising approaches for developing eco-friendly and highly effective biochemical agents.

In summary, addressing CR requires comprehensive, multidisciplinary innovative approaches that span the molecular to the field scale: from elucidating pathogen infection mechanisms and pathogen-host interactions, to harnessing wild resistance sources and optimizing microbiome-based interventions, each aspect plays a vital role. Through the integration of multi-omics analysis, genome editing, and predictive field modeling, durable wheat resistance to CR can be ultimately achieved.

## Data Availability

Not applicable.

## Abbreviations

CR: Crown rot
QTL: Quantitative trait locus
DON: Deoxynivalenol
NIV: Nivalenol
3-ADON: 3-Acetyldeoxynivalenol
15-ADON: 15-Acetyldeoxynivalenol
FDB: Fusarium detoxification of benzoxazolinone
RLK: Receptor-like kinase
WAK: Wall-associated kinase
JA: Jasmonic acid
SA: Salicylic acid
CTK: Cytokinin
IAA: Indoleacetic acid
IAAld: Indole-3-acetaldehyde
ROS: Reactive oxygen species
BOA: Benzoxazolinones
PGPR: Plant-growth-promoting rhizobacteria
Ag-NPS: Silver nanoparticles
Zn-NPS: Zinc nanoparticles
HHWGR: Huang-huai wheat-growing region
DEGs: Differentially expressed genes
DEPs: Differentially expressed proteins
TMT: Tandem mass tags
VIGS: Virus-induced gene silencing
FHB: Fusarium head blight
